# Balanced polyunsaturated fatty acids diet prevents the D-galactose-induced neuroinflammation and cognitive impairments

**DOI:** 10.1016/j.nsa.2026.106989

**Published:** 2026-02-12

**Authors:** Ivan Marniquet, Juliette Dupont-Viratelle, Flavie Crespo, Alexandra Séré, Sophie Layé, Jean-Christophe Delpech

**Affiliations:** University of Bordeaux, INRAE, Bordeaux INP, NutriNeuro, UMR 1286, F-33000, Bordeaux, France

**Keywords:** D-galactose model of accelerated aging, Neuroinflammation, Cognitive alterations, Polyunsaturated fatty acids, Anxiety

## Abstract

The rapid increase of the population aged 65 and over is associated with a higher prevalence of people suffering from cognitive functions alterations. Those impairments are partly due to environmental risk factors such as nutrition. In this context, nutrition, and more specifically the ratio of n-6/n-3 polyunsaturated fatty acids (n-6/n-3 PUFAs), has been extensively studied in relation to cognitive impairment and showed a potential beneficial effect on cognitive decline during aging. Clinical epidemiological studies showed a positive association between the n-3 PUFA consumption and cognitive abilities during aging. Preclinical studies have also demonstrated that dietary n-3 PUFA deficiency disrupts spatial memory, and induces alterations in neuronal functions and increased neuroinflammation, whereas dietary n-3 PUFA supplementation had beneficial effects on cognitive abilities and neuroinflammation in aged mice.

Here we showed that a balanced n-6/n-3 PUFAs precursors diet is sufficient to prevent the induction of accelerated aging characteristics such as contextual memory deficits, the altered emotionality status as well as the increased hippocampal neuroinflammation induced by D-galactose injections in mice, involving the activation of the AGE-RAGE pathway.

These results highlight the protective effects of a balanced n-6/n-3 PUFAs precursors on accelerated aging induced inflammatory, cognitive and emotional-like alterations.

## Highlights

• D-galactose model of accelerated aging induces neuroinflammation, emotional-like and cognitive alterations.
• Balanced n-6/n-3 PUFAs precursors diet prevents D-galactose-induced alterations.
• Long chain polyunsaturated fatty acids precursors could be used to prevent age-associated central alterations.

## 1 Introduction

Worldwide, our societies are facing a rapid increase in the population aged 65 and over, leading to a high number of individuals suffering from altered cognitive functions, affecting learning, working and episodic memory abilities (Deary et al., 2009). Aging is also associated with an increased risk to present alteration of the emotional status, including higher risk of anxiety disorders (Gulpers et al., 2016; Gao et al., 2023). These declines are often linked with a low-grade inflammation, or chronic inflammation, involving microglia, the main brain's immune cells. Indeed, previous work reported an increased production of inflammatory cytokines such as tumor necrosis factor-alpha (TNFa), Interleukin-6 (IL-6), and interleukin-12b (IL-12b) (Rafnsson et al., 2007; Franceschi and Campisi, 2014; Costa et al., 2021a). Chronic inflammation is currently considered as one of the 12 accepted hallmarks of aging (López-Otín et al., 2023). This enhanced production of pro-inflammatory cytokines in the brain, also called neuroinflammation, has been associated to neuronal loss (Zhang et al., 2024) and to an increased oxidative stress status and production of reactive oxygen species (ROS) (Rodrigues Siqueira et al., 2005; Swomley and Butterfield, 2015). The increased neuroinflammation in aging has also been related to the accumulation of Advanced Glycation End products (AGEs) at both the periphery and the brain in mouse and humans (Akhter et al., 2021; Vitorakis and Piperi, 2024), which can interact with his receptor RAGE and lead to cell signaling alterations, and increase of oxidative and inflammatory responses through the activation of the NF-kB pathway in peripheral macrophages in mouse and in central neurons in humans (Akhter et al., 2021; Bierhaus et al., 2005; Tóbon-Velasco et al., 2014; Jin et al., 2015).

The modulation of age-related cognitive decline and neuroinflammation has been an extensive topic of research in the recent decades, including multiple environmental factors such as nutrition. Throughout life, nutrition plays a part in establishing and maintaining an optimal cognitive health, both in rodents and humans, defined as our brain's ability to respond efficiently to any cognitive demand in order to limit cognitive decline (Song et al., 2022; Puri et al., 2023). Among nutrients, long chain polyunsaturated fatty acids (LC-PUFAs) have been widely studied in the context of aging (Joffre et al., 2020). Indeed, many clinical and pre-clinical studies showed the protective/preventive effect of n-3 LC-PUFAs supplementation on cognitive decline and anxiety (Vinot et al., 2011; Labrousse et al., 2012; Chataigner et al., 2021; Suh et al., 2024). They notably reported beneficial effects of n-3 LC-PUFAs onto spatial working memory associated to a reduction in the production of inflammatory cytokines in rodents (Delpech et al., 2015a; Rey et al., 2019; Joffre et al., 2019). One question arises in regard to the necessity to use a supplementation approach in n-3 LC-PUFA or if a balanced diet in LC-PUFA precursors could be sufficient to reduce cognitive decline during aging. We previously showed that a short-term exposition to such balanced LC-PUFA precursors diet in aged animals was not sufficient to prevent working memory deficits in mice (Labrousse et al., 2012; Moranis et al., 2012; Delpech et al., 2015b). We are now wondering if the use of such balanced diet in LC-PUFAs precursors during cognitive decline establishment could present a protective effect against aging-associated neuroinflammation, memory deficits and emotional-like alterations.

To address this question, we choose to use an accelerated aging model based on the systemic injection of D-galactose (D-gal) in mice. The D-gal model has been shown to induce subtle cognitive deficits and increased neuroinflammation similar to the one seen in aging (Song et al., 1999; Ross and Smith, 2020). At the mechanistic level, the D-gal-induced alterations have been linked to the activation of the AGE-RAGE pathway by promoting an accumulation of AGE in tissue at the periphery but also within the central nervous system leading to an artificial chronic increase of metabolic and oxidative stress (Haider et al., 2015; Yu et al., 2015). However, it is important to keep in mind that these cognitive deficits are induced by a chronic artificial increase of peripheral oxidative stress rather than a physiological, slow increase. Moreover, n-3 PUFAs supplementation decreased inflammation is associated with a decreased AGE-RAGE pathway activation in the central nervous system (Chen et al., 2017; Zhang et al., 2025). Previous work used this model to study the effect of n-3 LC-PUFAs on cognitive alterations. Indeed, a supplementation in fish oil or hydrolysate rich in n-3 LC-PUFAs in mice, has been shown to prevent spatial short- and long-term memory deficits associated with a reduction in the expression of proinflammatory markers in the hippocampus and AGE hippocampal accumulation (Zhang et al., 2022; Mougin et al., 2024; Martin et al., 2024). Here, we wonder if the use of a balanced n-6/n-3 PUFAs precursors diet in the D-gal model of accelerated aging could have the same preventive effects onto episodic-like memory or anxiety levels.

## 2 Methods

### 2.1 Animals

Male C57BL6/J mice (Janvier laboratories, Le Genest-Saint-Isle, France) were kept on a 12-h light/12-h dark schedule, housed on poplar woods chips litter in a controlled environment (21-23 °C, 40% humidity), with access to water and chow ad libitum. No statistical methods were used to predetermine sample size, and randomization of samples was done according to weight and adiposity. The mice were weighted three times per week from their arrival to their euthanasia. Adiposity was measured using NMR before the first day of injection and again before euthanasia. After the completion of behavioral testing, animals were euthanized, and tissues were collected and stored at −80 °C until analysis. All animal procedures followed the guidelines of the EU Directive 2010/63/EU for animal experiments and received approval from the national ethical committee for animal care and use (approval ID 39219). All data analysis was performed by researchers blinded to treatment groups.

### 2.2 Treatments and diets

Mice were divided into four groups equilibrated in weight and adiposity: saline with control diet (n = 10), D-gal-treated with control diet (n = 10), saline with balanced n-6/n-3 polyunsaturated fatty acids (PUFAs) diet (n = 10), and D-gal-treated with balanced n-6/n-3 diet (n = 10) (Table 1). Diets were provided by SAFE (SAFE laboratories, Augy, France). After more than one week of acclimatization, 10-week-old male mice were subjected to 10 weeks of the following experimental design: To induce accelerated aging model, mice received either subcutaneous injections of saline in the control group (0.9% NaCl (vol/vol) (saline groups); or received subcutaneous injections of 200 mg/kg of D-galactose dissolved in sterile 0.9% NacCl (vol/vol) (D-gal groups), with subcutaneously (s.c.) daily injections 6 out of 7 days per week (Zhang et al., 2022). The specific composition of the control (AO4) and balanced n-6/n-3 PUFAs diets are described in Table 1, values are coming from SAFE manufacturer (Aliments Scientifiques Premium). The lipid composition of the n-6/n-3 PUFA balanced diet was made to create a ratio of five n-6 PUFA to one n-3 PUFA at the precursors level, linoleic and alpha-linolenic acid respectively, in accordance with previous works (Labrousse et al., 2012; Delpech et al., 2015c; Joffre et al., 2016).

**Table 1:** Diets composition (with % of fatty acids) given by SAFE manufacturer.

Nutritional composition	*Percent of total diet (%)*	
	Control Diet (A04)	n-6/n-3 PUFAs Balanced diet
Nitrogen Free extract	60.4	70.62
*of wich Starch*	43.5	41.6
*of wich Sugars*	3.2	23
Crude Protein	16.1	15.87
Crude Fat	3.1	0.41
Crude Ash	4.6	4.12
Crude Fiber	3.9	1.48
Moisture	11.9	7.51

**Fatty Acids composition**	*Percent of total fatty acids (%)*	
	Control Diet (A04)	n-6/n-3 PUFAs Balanced diet
C16:0 Hexadecanoic acid (Palmitic acid)	21.3	21.4
C18:0 Octadecanoic acid (Stearic acid)	2.2	3.5
C16:1 (n-7) cis-Hexadec-9-enoic acid (Palmitoleic acid)	0.5	0.1
C18:1 (n-9) cis-Octadec-9-enoic acid (Oleic acid)	17.4	60.7
C18:2 (n-6) all-cis-9,12-octadecadienoic acid (Linoleic acid) LA	54.2	12.0
C18:3 (n-3) all-cis-9,12,15-octadecatrienoic acid (Alpha-linolenic acid (ALA))	4.3	2.3
**Ratio n-6/n-3 PUFAs**	**12.5**	**5.3**

We generated 4 groups: Control AO4 diet + Saline injection (Ctrl-Saline); Control AO4 diet + D-galactose injection (Ctrl-D-gal); Balanced n-6/n-3 PUFAs diet + Saline injection (Bal-Saline); Balanced n-6/n-3 PUFAs + D-galactose injection (Bal-D-gal).

### 2.3 Behavioral testing

All behavioral tests were performed in an empty testing room within the animal vivarium, during the light cycle between the hours of 9am-6pm. For all tests, animals were habituated in the test room for at least 1 h before the start of testing. A test-free period of 1-3 days was used between behavioral tests. The lighting, humidity, temperature, and ventilation were controlled and kept as constant as possible in the testing room. The experimenter was not present in the testing room during any of the tests. All behavioral analyses were performed in a blinded manner. Animal sample sizes are reported in the Figure legends.

#### 2.3.1 Novel object recognition

Animals were placed in a known open field (40 × 40 cm) in which two identical objects were placed. Interaction with the objects was manually and individually timed until reaching 20 s total at which the training phase was stopped as described previously (Leger et al., 2013). Mice that explored the objects less than 20 s total in 10 min were excluded. 24 h later, mice were place in the same environment but one object was replaced with a new object, the exploration was timed as during the training phase and same exclusion parameters. Random videos were timed again by a blinded experimenter to validate the results. An exploration ratio was calculated as the time spent with the new object over the total time of interaction, a ratio above 0.5 is indicative of a good recognition memory.

#### 2.3.2 Fear conditioning

Delayed fear conditioning was done during the light cycle following the protocol previously published (Suh et al., 2011). Briefly, on day 1, mice were placed in a context “A” and free to explore for a total of 600 s. At 240 s, a 20 s tone (90 dB, continuous white noise) was played and concomitant to the last 2 s, a mild foot shock at 0.5 mA was applied. This was repeated two more times at 380 s and 520 s. On day 2 (24 h later), mice were place in the same context “A” and allowed to explore for 300 s, without any tone or shock. On day 3 (24 h later), mice were place in a new context “B” and allowed to explore for 960 s. At 240 s, the same tone as day 1 was played for 60 s followed by 180 s of no-tone (post-tone period) but without shock. This was repeated two more time. In both contexts, the time of freezing behavior was recorded as a read-out of associative memory.

#### 2.3.3 Novelty suppress feeding (NSF)

After a 24-h food deprivation, mice were placed in a 34 × 50 × 30 cm box covered with unfamiliar beddings under a light with intensity >70 Lux and a pellet was centered in the arena. Each mouse spent 10 min in the arena and were tracked. The latency to eat was timed as an index of anxiety-like behavior. Directly after, mice were placed individually in a known cage and food consumption was monitored for 30 min to control for change in appetite and measured as the weight gain 30 min after the test.

#### 2.3.4 Open field (OF)

Mice were placed in an open chamber of 40 × 40 × 40 cm for 5 min under a light with an intensity >50 Lux at the center of the arena. Time and distance in the center (defined as 30 × 30 cm) and the periphery were tracked. We report here the time spent in the center and the total distance traveled as an indicator of the locomotor activity (Gould et al., 2009).

#### 2.3.5 Splash test

A fixed volume of water was sprinkled on the lower back of mice placed in a new cage (37 × 15 × 13cm) with clean bedding and light intensity <20 Lux. Grooming time was manually measured during 5 min as a proxy of agnosia and depressive-like-behavior.

### 2.4 Real-time quantitative PCR

The expression level of various target genes was assessed on hippocampus using real-time quantitative PCR. In brief, total RNAs were extracted from the hippocampus using RNeasy Plus Mini Kit (Qiagen, Germany), and the purity and quantity of RNA were measured for each sample using spectrophotometry (Nanodrop, Life Technologies, Saint Aubin, France). One micrograms of RNA were reverse transcribed into complementary DNA (cDNA) using Superscript III (Invitrogen, Life Technologies, Saint Aubin, France). Specific TaqMan primers were employed to amplify the genes of interest, focusing on IL-6 (Mm00446190_m1), IL-12a (Mm00434169_m1), TNF-α (Mm00443258_m1), IL-12b (Mm01288989_m1), RAGE (Mm00545815_m1) and IGF (Mm00439560_m1) for the hippocampus, with GAPDH (Mm99999915_g1) as the housekeeping gene. Fluorescence readings were recorded using a LightCycler 480 instrument II (Roche). Data were analyzed using the comparative threshold cycle (Ct) method, and the results were expressed as relative fold change to control target mRNA expression, following the approach outlined by Madore et al. (2020a).

### 2.5 Emotionality, cognitive and neuroinflammation Z-scores calculation

Z-scores are standardized by the group mean and group standard deviation and no normal assumption is made. Z-scores are mathematical tools allowing for mean-normalization of results within and across studies. Z= (x-μ)/σ represents the individual data for the observed parameter. μ and σ correspond to the mean and standard deviation of the control group respectively. In this study, the control diet with the saline treatment was defined as the control group for all the Z-scores: Associative, Emotionality and Inflammatory Z-scores.

The calculation of the emotionality Z-score was adapted from Guilloux et al. (2011) and corresponds to Z_OF_ - Z_NSF_. Briefly, the Z-score of the OF test normalized on peripheral locomotion was calculated as Z_OF_ = (Z_time_ (X = time in center) + Z_locomotion_ (X = distance traveled in periphery))/2, and the NSF Z-score corresponds to the delay for the animals to take a first bite (X = before first bite). A decrease of the emotionality Z-score corresponds to a negative emotional-like status (i.e. increased anxiety).

Associative memory Z-score was calculated as previously described (Mennesson et al., 2025). Briefly, the total associative memory Z-score consists of the mean of a contextual memory Z-score (where X = 100 – total freezing during contextual memory) and the tone/no-tone ratio in the cued memory test (where X = tone – ((habituation + post-tone)/2). A decrease in this Z-score corresponds to an altered associative memory with a modified contextual freezing response and decreased cued discrimination.

Finally, an inflammation Z-score was calculated as the addition of Z-score for each gene measured part of the inflammatory response (except RAGE). An increase in the inflammation Z-score corresponds to an increased neuroinflammation.

### 2.6 Statistical analysis

Data are presented as means ± standard error of the mean (s.e.m.). Comparison to a fixed-value was done using one-sample *t*-test. Comparisons between groups were done by either one-way or repeated measures 2-way or 3-way ANOVA, followed by a Fisher's LSD test *post hoc* test after assessing for data normality. Data analyses were performed using Prism 10.5 (GraphPad). A statistically significant difference was assumed starting at p < 0.05.

## 3 Results

### 3.1 Balanced n-6/n-3 PUFAs precursors diet prevents the deficit in associative memory induced by D-galactose

**Fig. 1:**
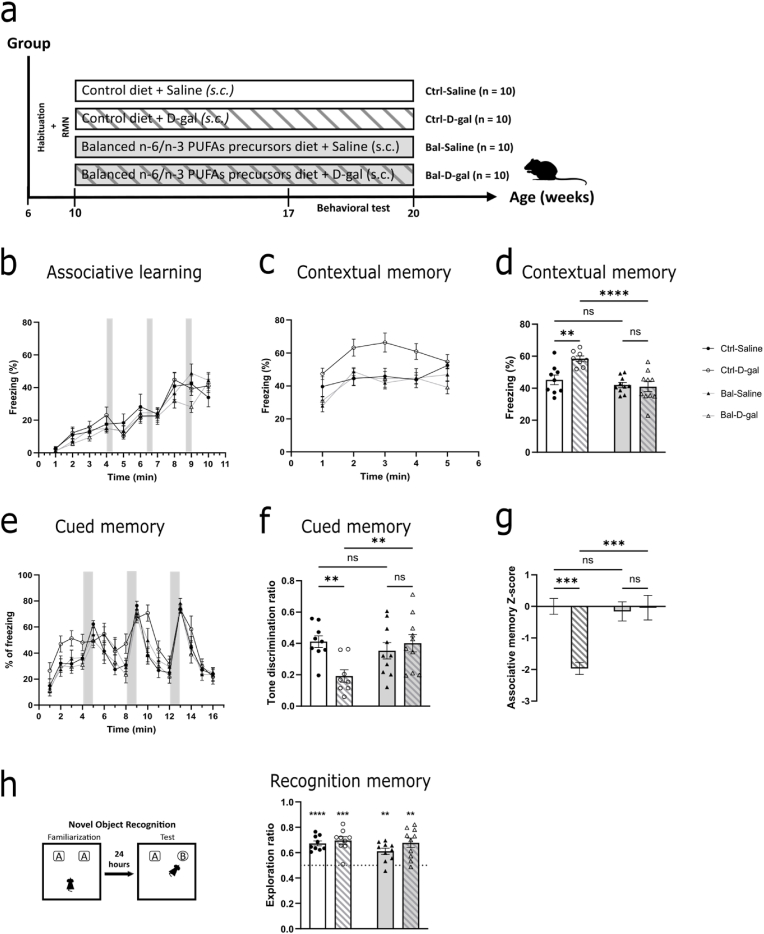
Balanced n-6/n-3 PUFAs precursors diet prevents the deficit in associative memory induced by D-galactose **(a)** Schematic representation of the four groups including Ctrl-Saline; Ctrl-D-gal; Bal-Saline; Bal-D-gal mice and the timeline of the experiment. **(b)** Time-course of freezing observed in the fear conditioning paradigm during training on day 1 showing no differences between the four groups (Three-way Anova on repeated measure interaction F (9, 132) = 1,276, p = 0,256). **(c)** Time-course of freezing observed during contextual-memory testing observed on day 2, showing a significant interaction between diet and D-gal treatment (Three-way Anova on repeated measure interaction F (1, 57) = 8,434, p < 0,01). **(d)** Average freezing time during contextual-memory testing observed on day 2, showing a significant interaction effect (Two-way Anova F (1,33 = 8,182, p < 0,01) with an increase in Ctrl-D-gal group (Fisher's LSD post-hoc: p < 0,01) significantly restored in Bal-D-gal group (Fisher's LSD post-hoc: p < 0,0001). **(e)** Time-course of freezing observed during cued-memory testing observed on day 3, showing a significant interaction between time, diet and D-gal treatment (Three-way Anova on repeated measure interaction F (15, 222) = 2,662, p < 0,001). **(f)** Tone discrimination index showing a significant interaction effect between diet and D-gal treatment (Two-way Anova interaction F (1,33) = 7,645, p < 0,01) with a significant decrease in discrimination index in Ctrl-D-gal group (Fisher's LSD post-hoc: p < 0,01) significantly restored in Bal-D-gal group (Fisher's LSD post-hoc: p < 0,01). **(g)** Associative memory Z-score showing a significant interaction between diet and D-gal treatment (Two-way Anova interaction F (1,33) = 11,47, p < 0,01) with a significant decrease in Ctrl-D-gal group (Fisher's LSD post-hoc: p < 0,001) significantly restored in Bal-D-gal group (Fisher's LSD post-hoc: p < 0,001). **(h)** (left) Novel object recognition test schematic representation. (right) Recognition memory abilities are not affected in any of the groups (One-sample *t*-test to chance level (50%) for Ctrl-Saline (t = 8,984, p < 0,0001); for Ctrl-D-gal (t = 6,615, p < 0,001); for Bal-Saline (t = 4,604, p < 0,01); for Bal-D-gal (t = 4,776, p < 0,01). N = 8-10 per group. Graph indicates mean ± s. e.m.

To determine the preventive effect of a balanced n-6/n-3 PUFAs precursors diet on cognitive dysfunction induced by D-gal, we compared balanced n-6/n-3 PUFAs precursors diet groups (Bal) to control AO4 diet groups (Ctrl) with or without D-gal treatment (Fig. 1A). Episodic-like memory in mice can be tested by measuring associative memory abilities (Kunčická et al., 2024). We thus tested the effect of a balanced n-6/n-3 PUFAs precursors diet on associative spatial memory using a delayed fear-conditioning paradigm (Suh et al., 2011). Mice in all four groups showed normal learning abilities during the acquisition phase on day 1 (Fig. 1B). On day 2, the contextual memory was tested and we found significantly increased fear memory in Ctrl-D-gal group, suggesting hippocampal-dependent cognitive alteration induced by D-gal treatment. Bal-D-gal group was strikingly similar to Bal-Saline group, and significantly reduced compared to Ctrl-D-gal group (Fig. 1C and D). These data indicate the potential preventive effect of a balanced n-6/n-3 PUFAs precursors diet onto D-gal-induced cognitive dysfunction. Cued memory, whose acquisition and consolidation is known to be regulated by the amygdala (Krabbe et al., 2018), was also affected by D-gal treatment with an interaction between time, diet and treatment. Indeed, we found a significant reduction in the discrimination index of the cued stimuli in the Ctrl-D-gal group compared to Ctrl-Saline group, which was absent in the Bal-D-gal group (Fig. 1E and F). Using a scoring approach combining contextual and cued memory responses, we found a significantly altered associative memory ability in the D-gal group compared to the Ctrl-Saline group and its absence in the Bal-D-gal group (Fig. 1G). Finally, we assessed recognition memory using novel object recognition with a 24 h interval. In all group, mice recognized the new object compared to chance level (Fig. 1H), indicating that neither the balanced n-6/n-3 PUFAs precursors diet nor the D-gal treatment have impacted the recognition memory.

### 3.2 Balanced n-6/n-3 PUFAs precursors diet prevents D-galactose-induced increases in anxiety-like behavior without affecting depression-like behavior

**Fig. 2:**
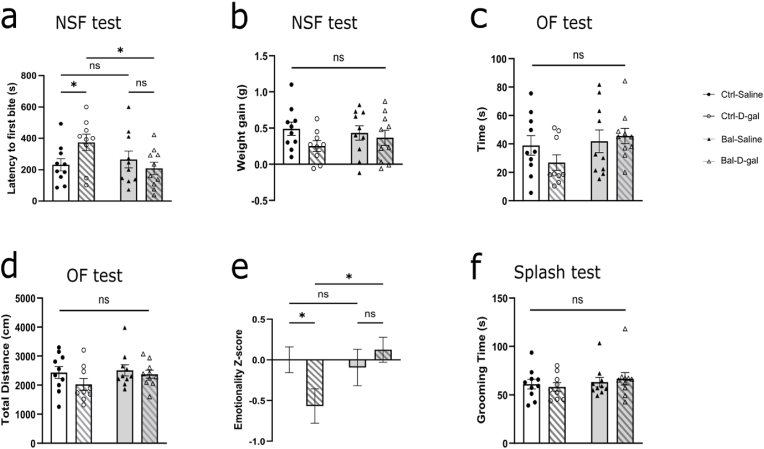
Balanced n-6/n-3 PUFAs precursors diet prevents the increased anxiety-like behavior induced by D-galactose. **(a)** Latency for the first bite observed in NSF test showing an interaction diet x treatment (Two-way Anova interaction F (1.35) = 4.602, p < 0.05). The increased latency observed in Ctrl-D-gal group (Fisher's LSD post-hoc: p < 0.05) is significantly restored in Bal-D-gal group (Fisher's LSD post-hoc: p < 0.05). **(b)** Weight gain 30 min after the test showing no differences between the four groups (Two-way Anova interaction F (1,35) = 0.8294, p = 0.3687). **(c)** Time spent in the center of the OF showing no differences between the four groups (Two-way Anova interaction F (1,35) = 1.446, p = 0.2373). **(d)** Total distance in the OF test showing no differences between the four groups (Two-way Anova interaction F (1,35) = 0.5471, p = 0.4645). **(e)** Emotionality Z-score showing a significant interaction between diet and D-gal treatment (Two-way Anova F (1,35) = 4.355, p = 0.0443) with a significant decrease in the Ctrl-D-gal group (Fisher's LSD post-hoc: p < 0.05) significantly restored in the Bal-D-gal group (Fisher's LSD post-hoc: p < 0.05) **(f)** Total grooming time during the splash test showing no differences between the four groups (Two-way Anova interaction F (1,35) = 0.3616, p = 0.5515). N = 9-10 per group. Graph indicates mean ± s. e.m.

To determine the status of anxiety-like behaviors following D-galactose treatment and its relationship to a balanced n-6/n-3 PUFAs precursors diet, we used NSF and OF tests. In the NSF test, Ctrl-D-gal group showed an increased delay in the first pellet-bite measurement compared to the Ctrl-Saline group. The altered delay was absent in the Bal-D-gal group and was similar to the Ctrl-Saline and Bal-Saline groups (Fig. 2A). To control for possible food consumption differences, a potential confounding factor to the NSF results, we measured body weight gain before and after the test. There were no differences in weight gain 30 min after the test in all four groups (Fig. 2B), suggesting that the balanced n-6/n-3 PUFAs precursors diet prevented the D-gal-induced increased anxiety-like behavior. In OF test, no difference in the time spent in the center (Fig. 2C), nor the total distance (Fig. 2D) between the four groups were found, indicating no locomotor alterations. Finally, taking into account both tests, we calculated an emotionality score as described previously (Guilloux et al., 2011). We found a reduced emotionality score in the Ctrl-D-gal group compared to the Ctrl-Saline group, absent in the Bal-D-gal group (Fig. 2E), indicating that the balanced n-6/n-3 PUFAs precursors diet could have prevented the emotional-like alterations linked to D-gal treatment.

We evaluated the depressive-like behavior in our model using the splash test. We could not find any significant differences between the four groups (Fig. 2F). Neither D-galactose treatment, nor the balanced n-6/n-3 PUFAs precursors diet significantly alter depressive-like behavior.

### 3.3 Balanced n-6/n-3 PUFAs precursors diet prevents the increased body adiposity induced by D-galactose

**Fig. 3:**
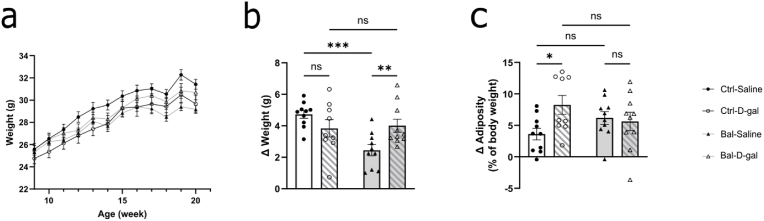
Balanced n-6/n-3 PUFAs precursors diet prevents the increased body adiposity induced by D-galactose. **(a)** Time course of the mice weight during the eleven weeks of diet and treatment intervention showing a significant interaction between time, diet and D-gal treatment (Three-way Anova repeated measure F (13,223) = 2.95, p < 0.001). **(b)** Weight gain during the diet and treatment procedure showing a significant interaction (Two-way Anova interaction F (1,35) = 9.349, p < 0.01) with a decreased weight gain in Bal-Saline group compared to Ctrl-Saline (Fisher's LSD post-hoc: p < 0.001) increased in the Bal-D-gal group (Fisher's LSD post-hoc: p < 0.01). No differences were observed between the Ctrl groups (Fisher's LSD post-hoc: p = 0.1317). **(c)** Adiposity changes during the diet and treatment intervention showing a significant interaction effect (Two-way Anova interaction F (1,35) = 4.519, p < 0.05) with a statistical increased in Ctrl-D-gal group compared to Ctrl-Saline (Fisher's LSD post-hoc: p < 0.05) and no differences between the Ctrl-Saline and Bal-Saline nor between the two Bal condition (Fisher's LSD post-hoc: p > 0.05). N = 9-10 per group. Graph indicates mean ± s. e.m.

We measured body weight throughout the experiment and we found an interaction between time, the diet and the D-gal treatment (Fig. 3A). We then calculated the body weight difference between the first and the last day of the experiment and we observed a weight gain significantly less important in the Bal-Saline group compared to Ctrl-Saline and a significantly higher weight gain in the Bal-D-gal group compared to the Bal-Saline group and no difference between the D-gal groups (Fig. 3B). To evaluate the body composition, we used NMR approach and revealed an increased adiposity in the Ctrl-D-gal group compared to the Ctrl-Saline group and no difference between the Bal-D-gal and the Bal-saline groups (Fig. 3C).

### 3.4 Balanced n-6/n-3 PUFAs precursors diet prevents the increased neuroinflammatory status induced by D-galactose

**Fig. 4:**
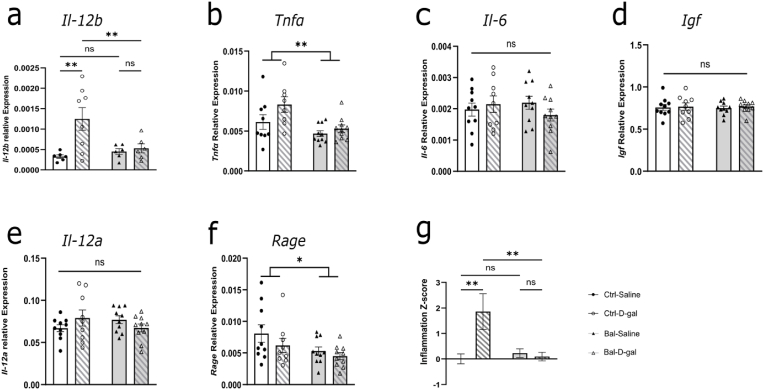
Balanced n-6/n-3 PUFAs precursors diet prevents the increased hippocampal neuroinflammatory status induced by D-galactose. (**a**) Relative gene expression of *Il-12b* with a significant interaction effect (Two-way Anova interaction F (1,22) = 5.266, p = 0.0317) with a significant increase in Ctrl-D-gal group (Fisher's LSD post-hoc: p < 0.01) corrected in the Bal-D-gal group (Fisher's LSD post-hoc: p < 0.01). **(b)** Relative gene expression of *Tnfα* with a significant diet effect (Two-way Anova diet effect F (1,33) = 10.38, p = 0.0029) and a tendency for treatment effect (Two-way Anova treatment effect F (1,33) = 4.105, p = 0.0509). **(c)** Relative gene expression of *Il-6* showing no statistical differences (Two-way Anova interaction F (1,35) = 1.711, p = 0.1993). **(d)** Relative gene expression of *Igf* showing no statistical differences (Two-way Anova interaction F (1,35) = 0.03343, p = 0.856). **(e)** Relative gene expression of *Il-12a* showing no statistical differences (Two-way Anova interaction F (1,35) = 3.369, p = 0.0749). **(f)** Relative gene expression of *Rage* showing a significant diet effect (Two-way Anova diet effect F (1,35) = 5.161, p < 0.05). **(g)** Inflammatory score calculated as described in the methods showing a significant interaction effect (Two-way Anova interaction F (1,35) = 7.264, p < 0.01) with a statistically increased inflammatory score in the Ctrl-D-gal group (Fisher's LSD post-hoc: p < 0.01) corrected in the Bal-D-gal group (Fisher's LSD post-hoc: p < 0.01). N = 6-10 per group. Graph indicates mean ± s. e.m.

Previous work reported an increased inflammatory status in the brain of D-galactose-treated animals (Yu et al., 2015; Gao et al., 2015; Rehman et al., 2017). We evaluated the ability of the balanced n-6/n-3 PUFAs precursors diet to prevent its induction by D-galactose treatment. We analyzed the expression of genes linked to neuroinflammation, including *Tnfα*, *Il-6*, *Il-12a-b*, *Igf* (Shi et al., 2020; Rauf et al., 2022). D-galactose injections significantly increased the expression of *Il-12b* in the Ctrl-D-gal group compared to the control group and this effect was prevented by the balanced n-6/n-3 PUFAs precursors diet. Indeed, the Bal-D-gal group showed significantly reduced levels compared to Ctrl-D-gal group (Fig. 4A). For *Tnfα*, we only saw a significant diet effect with a trend for a D-gal effect (Fig. 4B). For the other inflammatory gene expression tested, we did not have any significant effects. (Fig. 4C to E). We also measured RAGE level as a proxy of D-galactose treatment. Interestingly, *Rage* expression was decreased by the balanced n-6/n-3 PUFAs precursors diet compared to the control diet, but there was no effect of the D-galactose injection (Fig. 4. F). Finally, to consider possible individual variations to nutritional intervention, an Inflammation Z-score was calculated taking into account the cytokines expression measured in this study. We found an increased inflammation score in the Ctrl-D-gal group compared to the Ctrl-Saline group and absent in the Bal-D-gal group (Fig. 4G), indicating that the balanced n-6/n-3 PUFAs precursors diet may have prevented the increased inflammatory status induced by D-gal treatment.

## 4 Discussion

Aging is a physiological occurring mechanism that can be modulated by numerous environmental factors and represents a major challenge for the health system with the increased proportion of elderly within the total population. It is thus important to better understand and refine strategies known to alleviate age-related cognitive alterations. As neuroinflammation is a common hallmark in physiological aging (Franceschi and Campisi, 2014) and is related to cognitive decline (Rafnsson et al., 2007), it seems crucial to dampen it in order to promote healthy aging. Among the causes for this increased neuroinflammation, AGEs accumulation is known to participate in the age related cognitive decline by promoting inflammation in the central system but also at the periphery by its interaction with his specific receptor RAGE (Vitorakis and Piperi, 2024; Tóbon-Velasco et al., 2014; Ross and Smith, 2020). Among nutrients, LC-PUFAs have been shown to reduce neuroinflammation and supplementation with LC-PUFA has been shown to improve cognitive abilities during aging (Chataigner et al., 2021).

In this study, our aim was to investigate the protective effect of a balanced n-6/n-3 PUFAs precursors intake on the emotional-like behavior, neuroinflammatory, and subtle cognitive alterations found in an accelerated model of aging (D-gal model). We showed that a balanced intake of n-6/n-3 PUFAs (5:1) precursors starting at the induction of the D-gal model, seems to be sufficient to prevent the cognitive, emotional-like behavior, adiposity and neuroinflammatory-related alterations in the hippocampus induced by systemic D-gal injections. Indeed, a balanced n-6/n-3 PUFAs precursors diet prevented associative memory impairment in both contextual and cued memory, while also correcting the increased anxiety-like behavior. Additionally, the global neuroinflammatory status is also maintained to normal by the n-3 PUFAs balanced diet with D-gal.

In regard to cognitive abilities and its relationship to nutrition, the model of D-gal accelerated aging has been previously used in multiple studies to understand the mechanism of protection by specific nutrient against age-related cognitive impairments. Cumulative studies have demonstrated the protective effect of n-3 LC-PUFAs supplementation or n-3 PUFA increase in detriment of n-6 PUFAs intake during 10 weeks on the spatial short and long term cognitive impairments associated with a reduction in some inflammatory markers (Zhang et al., 2022; Mougin et al., 2024; Martin et al., 2024). Another group demonstrated that both n-3 LC-PUFAs supplementation and a balanced n-3 PUFAs intake during 3 month after 5 month of D-gal injection in a diabetic rat model can restore spatial learning and decrease *Rage* expression in the pre-frontal cortex, but only the balanced n-6/n-3 PUFAs intake restored the spatial memory, not the n-3 LC PUFAs supplemented group (Guo et al., 2018). However, in our hand, the D-galactose treatment did not impaired recognition memory compared to the literature, a possible explanation could be that the mnemonic demand, representing the task difficulty, was too low in our case. Future research could test whether an increased retention time or more similar objects, two parameters capable of increasing the mnemonic demand in this test, could show a deficit in recognition memory in the Ctrl-D-gal group. Another explanation would be that recognition memory compared to spatial memory does not rely solely on hippocampus but it's connection with other structure such as the pre-frontal cortex and perirhinal cortex (among others) (Broadbent et al., 2004; Winters et al., 2004), that might be less altered in our hand as in other articles using this D-gal approach (Wang et al., 2009). Additionally, the D-gal treatment induced an associative memory alteration with an increased freezing to the context and decreased cued discrimination. This effect of D-gal treatment was overall clearly visible in the associative memory score. Interestingly, the n-6/n-3 PUFAs precursors balanced diet was sufficient to correct those D-gal-induced associative memory alterations, in line with previous research showing a beneficial effect of n-3 LC-PUFAs supplementation onto associative memory measured using the Morris water maze test in aged mice (Chataigner et al., 2021; Joffre et al., 2020). Similarly, beneficial effects in aged population were reported after a supplementation in n-3 LC-PUFAs onto episodic memory deficits in humans (Petersson and Philippou, 2016; Yurko-Mauro et al., 2015).

The D-gal model has also been shown to increase anxiety like behavior in rodent (Haider et al., 2015; Hakimizadeh et al., 2021). However, no studies have shown the emotional-like behavior associated with PUFAs intake in this model. Our study is the first to report that a balanced n-6/n-3 PUFAs precursors intake in the D-gal model may prevent the altered emotional-like status by decreasing the delay to first bite in NSF test and restoring the emotionality score. Noteworthy, as this model is based on daily *s. c.* injection, the over manipulation of the mice could have led to a disinterest of behavioral test in which no strong novelty or aversive stimulus was present. To discard this possible depressive-like factor, we showed with the Splash test that animals had a normal grooming behavior, indicating no signs of depressive-like-behavior. Knowing that previous work reported a positive effect of n-3 PUFAs on the hypothalamic-pituitary-adrenal (HPA) axis and its dysregulation in aging and cognitive impairment, it could have been of interest to access the level of BDNF and the stress hormone corticosterone (Rodrigues Siqueira et al., 2005; Kim et al., 2020).

In regards to the inflammatory status, we found a beneficial effect of the balanced n-6/n-3 PUFAs precursors diet onto the hippocampal neuroinflammatory status in the D-gal model. As aging is associated with a chronic low grade inflammation in the whole body (Li et al., 2023a), it could have been of interest to test whether the D-gal treatment could induce an impaired response to peripheral LPS-induced inflammation, absent in the balanced n-6/n-3 PUFAs animals. Indeed, previous study has shown that n-3 PUFAs participate in those processes by promoting the resolution of inflammation (Delpech et al., 2015c). Interestingly, the balanced n-6/n-3 PUFAs precursors intake did reduce the expression of *Rage* in the hippocampus, representing a potentially relevant mechanism underlying the protection against neuroinflammation. This effect on *Rage* expression was absent in other studies using supplementation in n-3 LC-PUFAs rising the question of the use of n-3 PUFAs precursors diet as a long-term protective effect. Nonetheless, further research is needed to confirm the possible direct effect of n-6/n-3 PUFAs precursors on *Rage* expression, its validation at the protein level, and the addition of microglial phenotypic measurements (Madore et al., 2020b; Costa et al., 2021b).

Another limitation is the lack of exploration of the mitochondria. Indeed, previous work showed that n-3 LC-PUFAs and D-gal administration can affect mitochondrial functions by modulating super-oxide dismutase expression or impair mitochondrial structure respectively (Shi et al., 2020; Ferreira et al., 2015; Hernando et al., 2019). Mitochondria dysfunction is also one of the 12 hallmarks of aging (Li et al., 2023b). Thus, future research is needed to assess mitochondrial functions at the structural level in this model in relation to n-6/n-3 PUFAs precursors balanced diet preventive strategy. Finally, another important limitation in this study is the use of only male mice knowing that sexual heterogeneity exists in both PUFAs metabolism and RAGE activation (Du et al., 2022; Lamontagne-Kam, 2023). Future research is needed using this paradigm applied to female mice to assess the ability of a balanced n-6/n-3 PUFAs precursors diet to prevent the D-gal-induced impairments.

Even though this study showed the preventive effect of a balanced n-6/n-3 PUFAs precursors intake on three known dimensions impacted by aging (i.e. cognition, emotional-like behavior and neuroinflammation), one clear limitation is the use of an accelerated model where the AGE-RAGE pathway is overactivated. The fact that we found a beneficial effect by using a balanced diet in n-6/n-3 PUFAs precursors instead of a supplementation in LC-PUFAs, similar to the results found by Guo and collaborators (Guo et al., 2018), reinforce the need to continue to document the beneficial effect of a balanced n-6/n-3 PUFAs precursors diet onto aging by testing it in a more physiological aging model. Importantly, our strategy focused on the use of an n-6/n-3 PUFAs precursors balanced diet instead of a supplementation approach, carries a strong potential to be valorized as a relevant source of information in the context of public health prevention strategies for age-related alterations.

## Author contributions

Conceptualization, J.C.D; Methodology, J.C.D., I.M., J.D.V; Investigation, I.M., J.D.V., F.C.; Writing – Original Draft, J.C.D.,I.M.; Writing – Review & Editing, all authors; Formal Analysis: J.C.D., I.M., J.D.V.; Funding Acquisition, J.C.D., S.L.; Supervision, J.C.D.

## Funding

This work was funded in part by the Région Nouvelle-Aquitaine CHESS Exomarquage 13059720-13062120 (JCD, SL), the JPND SOLID ANR-21-JPW2-0004-05 (SL, JCD), the ANR-23-CE14-0056-01 (JCD), and the French National Ministry of Research and Higher Education (IM).

## Conflict of interest

In regards to the full research article entitled “Balanced polyunsaturated fatty acids diet prevents the D-galactose-induced neuroinflammation and cognitive impairments” submitted to your journal, I declare no conflict of interest.
